# Synthetic versions of firefly luciferase and *Renilla* luciferase reporter genes that resist transgene silencing in sugarcane

**DOI:** 10.1186/1471-2229-14-92

**Published:** 2014-04-08

**Authors:** Ting-Chun Chou, Richard L Moyle

**Affiliations:** 1 School of Agriculture and Food Sciences, University of Queensland, Brisbane 4072, Australia

## Abstract

**Background:**

Down-regulation or silencing of transgene expression can be a major hurdle to both molecular studies and biotechnology applications in many plant species. Sugarcane is particularly effective at silencing introduced transgenes, including reporter genes such as the firefly luciferase gene.

Synthesizing transgene coding sequences optimized for usage in the host plant is one method of enhancing transgene expression and stability. Using specified design rules we have synthesised new coding sequences for both the firefly luciferase and *Renilla* luciferase reporter genes. We have tested these optimized versions for enhanced levels of luciferase activity and for increased steady state luciferase mRNA levels in sugarcane.

**Results:**

The synthetic firefly luciferase (luc*) and *Renilla* luciferase (Renluc*) coding sequences have elevated G + C contents in line with sugarcane codon usage, but maintain 75% identity to the native firefly or *Renilla* luciferase nucleotide sequences and 100% identity to the protein coding sequences.

Under the control of the maize pUbi promoter, the synthetic luc* and Renluc* genes yielded 60x and 15x higher luciferase activity respectively, over the native firefly and *Renilla* luciferase genes in transient assays on sugarcane suspension cell cultures.

Using a novel transient assay in sugarcane suspension cells combining co-bombardment and qRT-PCR, we showed that synthetic luc* and Renluc* genes generate increased transcript levels compared to the native firefly and *Renilla* luciferase genes.

In stable transgenic lines, the luc* transgene generated significantly higher levels of expression than the native firefly luciferase transgene. The fold difference in expression was highest in the youngest tissues.

**Conclusions:**

We developed synthetic versions of both the firefly and *Renilla* luciferase reporter genes that resist transgene silencing in sugarcane. These transgenes will be particularly useful for evaluating the expression patterns conferred by existing and newly isolated promoters in sugarcane tissues. The strategies used to design the synthetic luciferase transgenes could be applied to other transgenes that are aggressively silenced in sugarcane.

## Background

Molecular analysis of transgene expression can be hampered by events that lead to erratic, diminished or absolute loss of expression [1,2]. Early attempts to express foreign transgenes frequently resulted in low levels of protein accumulation in transgenic plants. Vaeck et. al. introduced the *bt2* gene from *Bacillus thuringiensis* into tobacco and detected the expressed protein at only 0.0002-0.02% of total soluble protein [3]. Furthermore the majority of the *bt2* transcripts were shorter than expected, presumed to be due to premature polyadenylation [4] and/or posttranscriptional processing and transcript instability [5]. Similarly, the GFP reporter gene from *Aequorea victoria* is not expressed well in certain plant species. Little or no GFP fluorescence could be detected in *Arabidopsis* or tobacco transgenic plants despite the use of strong promoters such as CaMV 35S [6,7].

Plant coding sequences generally have a codon bias relatively high in G + C content compared to bacteria, insects and other sources of foreign transgenes. A lower percentage G + C content and associated codon bias in foreign transgenes appears to have a major influence on transgene expression. Low transgene expression correlating with differences in codon bias has been hypothesised to be due to lower availability of specific tRNAs encoded by rare codons. The presence of these rare codons may cause stalling during translation, thereby destabilizing the transcripts by leaving the mRNA exposed to components of the RNA degradation machinery. Furthermore, A + T rich transgene sequences may provide motifs that will function as splice sites, polyadenylation sequences and RNA destabilizing elements in plants [4,8,9]. Thus using the redundancy of the genetic code to design synthetic copies of foreign transgenes with higher G + C contents can potentially not only increase translational efficiency, but can also remove deleterious A + T rich sequence motifs responsible for mRNA instability [1]. Both GFP and *bt2* transgene expression were greatly enhanced in transgenic plants when the codon usage was optimized, increasing the overall G + C content [5,7]. A modified firefly luciferase gene with improved codon usage for mammalian cells (1.8% higher G + C content) had 14-23× increased activity in maize suspension cells and 53-59× increased activity in wheat scutellum [10].

Sugarcane species are tall perennial grasses of the genus *Sacharrum*. The jointed fibrous sugarcane stalks are rich in sugar and can measure up to six meters tall. Modern cultivars are highly polyploidy hybrids of *S. officinarum* and *S. spontaneum* with *2n* = 100–130 chromosomes [11]. Sugarcane is an important economic crop, responsible for the majority of the world’s sugar production and is also recognised as the most sustainable of the current generation of biofuel crops. As such there is a large degree of interest in researching and engineering sugarcane varieties using molecular and biotechnology approaches [12]. Substantial progress has been made to develop the enabling technologies and tools necessary for molecular analysis and applied biotechnology in sugarcane. An extensive EST collection has been amassed [13], small RNA developmental profiles analyzed [14-17], (Sternes and Moyle: Deep sequencing reveals divergent expression patterns within the small RNA transcriptomes of cultured and vegetative tissues of sugarcane, under review) transformation systems developed [18,19] and promoter sequences isolated [[20-23,24]]. However, molecular analysis in transgenic sugarcane has been hampered by events that have led to erratic, diminished or absolute loss of foreign transgene expression. Past studies aimed at the functional analysis of promoter sequences targeted for use in sugarcane biotechnology have reported aggressive silencing of reporter transgenes. Expression of the GUS reporter gene under the control of the ubi4 or ubi9 promoter was aggressively repressed in transgenic sugarcane plants [25]. Nuclear run-off assays prove the repression of the GUS transgene expression was due to post-transcriptional silencing (PGTS) [25]. Mudge et al. (2009) [24] reported expression of both the GUS-*Plus* and firefly luciferase reporter genes were similarly repressed in mature sugarcane plants, even under the control of endogenous MYB gene promoters. Indeed the firefly luciferase gene was found to be strongly down-regulated in transgenic sugarcane lines under the control of a range of endogenous, foreign and recombinant promoters [26,27]. Recently a synthetic GUS transgene, codon optimised for use in sugarcane, was shown to generate significantly higher levels of GUS activity than the native GUS reporter gene [21]. Similar design rules were applied to synthesize a silencing resistant version of a sucrose isomerase transgene, used in metabolic engineering of sugarcane to produce alternative sucrose isomers [19,28].

Firefly luciferase has been used as a reporter gene of promoter function in many plant species [29-35]. The luciferase reaction emits light that can be detected under long exposure camera images or quantified using a luminometer. Together with the *Renilla* luciferase gene, dual luciferase assays are particularly useful in promoter analysis studies, where the *Renilla* luciferase gene is typically used to normalise expression across different co-bombardments, transfections or treatments [35,36]. However, the propensity for firefly luciferase transgene expression to be aggressively repressed in transgenic sugarcane is a major obstacle to the usefulness of luciferase as a reporter of promoter function in sugarcane [24].

We have investigated designing synthetic versions of both the firefly luciferase and *Renilla* luciferase reporter transgenes as a strategy to combat silencing mechanisms in sugarcane. By synthesising a higher G + C content in line with sugarcane codon usage and avoiding known RNA instability motifs, we successfully designed firefly and *Renilla* luciferase transgenes that yield significantly higher activity levels in both transient assays and in stable transgenic lines of sugarcane. Additionally, we used a novel transient co-bombardment and quantitative real time PCR assay to determine that the increase in expression from the optimised luciferase transgenes is at least in part due to increased mRNA stability.

## Results

### Sequence analysis of synthetic firefly and *Renilla* luciferase coding sequences

The average G + C content of 71 *Sacchurum officinarum* gene coding sequences in the Kazusa database is 55.7% [37]. In contrast, firefly luciferase (*luc*) and *Renilla* luciferase (Ren*luc*) coding sequences have G + C contents of just 45% and 36.5% respectively. We took advantage of the redundancy of the genetic code to design new sequences encoding for synthetic versions of *luc* and Ren*luc*, and to eliminate rare codons, strings of A or T bases, palindromes, polyadenylation signals, and to avoid the potential addition of an intron (refer to Methods section for details). The synthetic versions, denoted as luc* and Renluc*, have a G + C content of 55% and 52% respectively. Sequence alignment reveals the synthetic luc* and Renluc* sequences share 75% identity with the native *luc* and Ren*luc* sequences (Additional file 1: Figure S1 and Additional file 2: Figure S2). The sequence of luc* and Renluc* can be found in GenBank accessions KC147725 and KJ140114, respectively.

### luc* and Renluc*generate higher transient expression levels than the native firefly and *Renilla* luciferase genes

**Figure 1:**
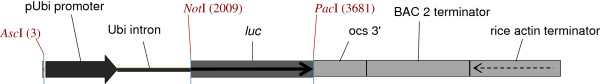
**The pUbi-*luc *construct.** The luc*, Ren*luc* and Renluc* coding sequences were also cloned into the pUbi T3 backbone vector using the unique *Not*I and *Pac*I restriction enzyme sites.

The native *luc* and Ren*luc* coding sequences, and the synthetic luc* and Renluc* coding sequences were each cloned into the pUbi T3 expression plasmid backbone (Figure 1). Transient expression from the synthetic versions was compared to that from the native luciferase genes using the dual luciferase assay, in plated sugarcane suspension cells.

**Figure 2:**
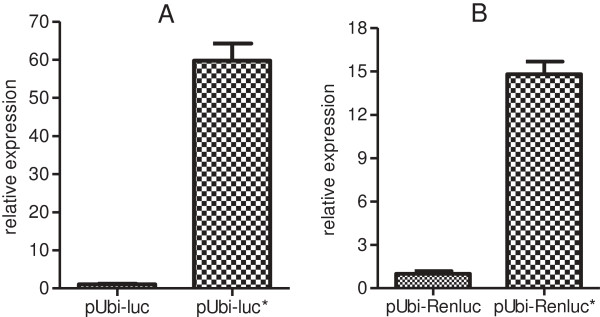
**Dual Luciferase assays in sugarcane suspension cells. A**. Equimolar amounts of pUbi-*luc* or pUbi-luc* were co-bombarded with equimolar amounts of pUbi-Renluc*. After using pUbi-Renluc* activity to normalise expression between bombardments, the results show on average 60-fold higher luciferase activity generated from the synthetic luc* transgene relative to the native firefly *luc* transgene. **B**. Equimolar amounts of pUbi-Ren*luc* or pUbi-Renluc* were co-bombarded with equimolar amounts of pUbi-luc*. After using pUbi-luc* activity to normalise expression between bombardments, the results show on average 15-fold higher luciferase activity generated from the *Ren*luc* transgene relative to the native Ren*luc* transgene. Error bars represent the standard error of the mean from four co-bombardments of sugarcane suspension cells.

Using the pUbi*-*Renluc* construct to normalise firefly luciferase expression across each co-bombardment, dual luciferase activity measurements revealed the pUbi-luc* construct produced on average 60-fold higher luciferase activity than the pUbi-*luc* construct (Figure 2A and Additional file 3: Figure S3).

Using the pUbi-luc* construct to normalise *Renilla* luciferase expression across each co-bombardment, dual luciferase activity measurements showed the pUbi-Renluc* construct produced on average 15-fold higher luciferase activity than pUbi-Ren*luc* (Figure 2B).

### Synthetic luc* and Renluc* generate higher steady state transcript levels than the native *luc* and *Renluc* transgenes

**Figure 3:**
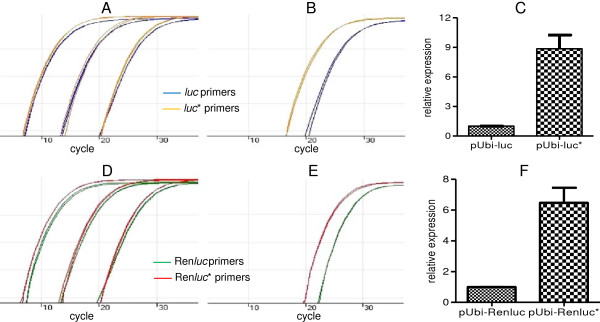
Co-bombardment and qRT-PCR assays reveal the synthetic luc* and Renluc* transgenes produce increased levels of steady state mRNA relative to the native luciferase transgenes. A. qRT-PCR on equimolar serial dilutions of pUbi-*luc* (orange lines) and pUbi-luc* (blue lines) plasmid template using the corresponding gene-specific primer pair (Additional file 4 Figure S4A&B). **B**. Amplification from cDNA synthesised from suspension cells co-bombarded with equimolar amounts of pUbi-*luc* and pUbi-luc* plasmid. **C**. luc* transcript levels were on average 8.9-fold higher than *luc* transcript levels in the co-bombarded suspension cells cDNA. Error bars represent the standard error of the mean across three independent co-bombardments. **D**. qRT-PCR on equimolar serial dilutions of pUbi-Ren*luc* (green lines) and pUbi-Renluc* green lines) plasmid using the corresponding gene specific primer pair (Additional file 4: Figure S4A&B). **E**. Amplification from cDNA synthesised from suspension cells co-bombarded with equimolar amounts of pUbi-Ren*luc* and pUbi-Renluc* plasmid. **F**. Synthetic Renluc* transcript levels were on average 6.5-fold higher than the native Ren*luc* transcript levels in the co-bombarded suspension cells cDNA. Error bars represent the standard error of the mean across three independent co-bombardments.

It is of interest to determine if the increase in luciferase activity attributed to the synthesized luc* and Renluc* genes is due solely to enhanced translational efficiency or if there is an increase in the stability of the mRNA levels. To quantify relative steady state mRNA levels generated from each transgene, we devised a novel co-bombardment and quantitative real time PCR (qRT-PCR) assay. We took advantage of nucleotide differences between the *luc* and luc* transgenes to design gene specific qRT-PCR primers that amplify equal lengths of each target sequence from the 3’ region of each transgene with equal efficiency (Additional file 4: Figure S4). The ability of the *luc* and luc* primer pairs to amplify at even rates was tested on serial dilutions of equimolar amounts of pUbi-*luc* or pUbi-luc* template (Figure 3A). Using the same approach, the Ren*luc* and Renluc* primer pairs were also shown to amplify at an even rate (Figure 3D).

Equal quantities of pUbi-*luc* and pUbi-luc* (or pUbi-Ren*luc* and pUbi-Renluc*) plasmid were subsequently co-bombarded at sugarcane suspension cell cultures. RNA was extracted 24 h post-bombardment and cDNA synthesised. qRT-PCR reactions using the cDNA as a template revealed a shift in the amplification curve between reactions using either the native *luc* or synthetic luc* primer pairs (Figure 3B and E). As each primer pair amplifies at an equal rate, the shift in the amplification curve is due to a differential abundance of *luc* transcript relative to the luc* transcript in the co-bombarded suspension cell cDNA. The co-bombardment and qRT-PCR assay reveals ~9-fold higher luc* transcript levels, on average, relative to *luc* (Figure 3C). Using the same co-bombardment and qRT-PCR approach, we detected ~6.5-fold higher Renluc* transcript levels, on average, relative to Ren*luc* (Figure 3F). This co-bombardment and qRT-PCR approach assumes there is no gene specific bias during cDNA synthesis.

### The synthetic luc* transgene generates substantially higher luciferase activity than the native firefly *luc* transgene in stably transformed lines

**Figure 4:**
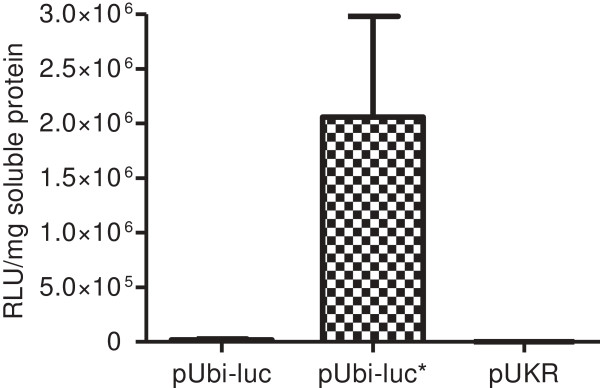
**Luminometer assays on plantlets regenerated from transgenic calli.** Across 15 transgenic lines, the pUbi-*luc* plantlets had an average luminometer relative light units (RLU) reading of 20043 per mg of soluble protein. Across 15 transgenic lines, the pUbi-luc* transgenic plantlets had an average RLU of over 2205284 per unit soluble protein, representing an on average 110-fold increase over pUbi-*luc* lines. The 11 pUKR negative control transgenic lines yielded no detectable luciferase activity. The error bars represent the standard error of the mean.

Sugarcane cultivar Q117 calli were transformed with either pUbi-*luc* or pUbi-luc* plasmid via co-bombardments with the previously described pUKR plasmid containing an nptII selectable marker [18]. Luminometer based assays on whole plantlets regenerated from 15 pUbi-*luc* and 15 pUbi-luc* transgenic lines revealed on average 110× higher luciferase activity in the pUbi-luc* lines than in the pUbi-*luc* lines (Figure 4). There was no significant difference in the average transgene copy number between pUbi-*luc* and pUbi-luc* populations of transgenic lines (Additional file 5: Figure S5).

**Figure 5:**
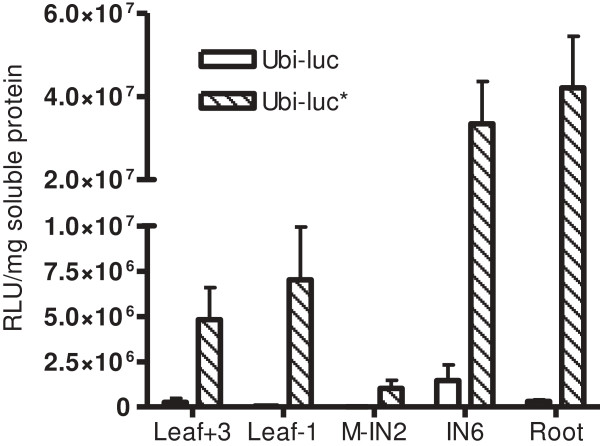
Luminometer assays on tissue extracts from pUbi-*luc *and pUbi-luc* maturing plant transgenic lines. Glasshouse grown plants containing approximately 20 internodes (~1.8-2.1 m tall to the uppermost visible dewlap) were harvested after approximately 10 months of growth and tissue extracts assayed for luciferase activity. M-IN2 represents tissue containing the meristem down to internode 2. IN6 represents internode 6 tissue. Empty fill bars represent data from pUbi-*luc* lines whereas cross fill bars represent data from pUbi-luc* lines. RLU stands for relative light units, the output measurement of the luminometer. The error bars represent the standard error of the mean across 15 transgenic lines per construct.

**Figure 6:**
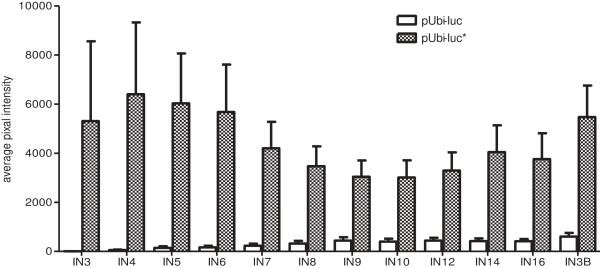
Camera assays on stem internode sections from pUbi-*luc *and pUbi-luc* maturing transgenic plant lines. Assayed glasshouse grown plant stems typically contained ~20 internodes (~6-7 ft tall to the uppermost visible dewlap). The average pixel intensities were background subtracted. IN3B represents the third internode from the base of the stem. The error bars represent the standard error of the mean across 15 transgenic lines per construct.

Transgenic lines were also assayed for luciferase activity at the ~20 internode (IN) stage of development (~1.8-2.1 m tall to the top visible dewlap). Luminometer assays showed significantly higher luciferase activity that was generated from the pUbi-luc* lines compared to the pUbi-*luc* lines in each tissue tested (Figure 5). A camera based luciferase assay on internode sections across the stem profile for each of the aforementioned 20 IN stage of development lines further confirms significantly higher luciferase activity in pUbi-luc* lines compared to pUbi-*luc* lines (Figure 6).

Interestingly, the fold increase in luciferase activity attributable to the pUbi-luc* construct varied substantially from tissue to tissue (Table 1). Between IN 9 and IN 16 there was, on average, less than 10-fold difference in luciferase activity generated from pUbi-*luc* and pUbi-luc* lines. However, the difference trended substantially higher in younger internode tissues. For example the fold difference between the mean luciferase activity was over 800-fold at IN 3 tissue compared to less than 10× in the mature internodes (Additional file 6: Table S1). The meristem-IN 2 tissue sample exhibited the largest fold difference, with luciferase activity estimated at over 7000× higher, on average, in the pUbi-luc* population of lines. Similarly, the younger leaf -1 tissue exhibited a higher fold difference in average luciferase activity than the older leaf +3 tissue (Table 1).

**Table 1:** **Fold expression changes in multiple tissues assayed from pUbi-*luc *** and pUbi-luc*  **transgenic lines of sugarcane**

**Luminometer assay**	**pUbi-*luc *** **Mean +/- SD**	**pUbi- *luc *** * Mean +/- SD	**Mean fold difference**
Plantlets	20040 +/- 8311	2215000 +/- 979000	110x
Leaf +3	264000 +/- 205500	4829000 +/- 1770000	18x
Leaf -1	52730 +/- 23730	7030000 +/- 2913000	133x
Meristem-Internode 2	139.4 +/- 105.8	1031000 +/- 447200	7396x
Internode 6	1455000 +/- 869400	33430000 +/- 10180000	23x
Root	302800 +/- 82140	42080000 +/- 12360000	139x
**Camera assay**			
Internode 3	6.619 +/- 3.353	5312 +/- 3249	802x
Internode 4	56.70 +/- 19.09	6430 +/- 2925	113x
Internode 5	144.3 +/- 64.86	6033 +/- 2029	42x
Internode 6	165.2 +/- 68.15	5680 +/- 1937	34x
Internode 7	231.6 +/- 85.04	4203 +/- 1077	18x
Internode 8	323.6 +/- 109.7	3470 +/- 813.3	11x
Internode 9	440.3 +/- 137.4	3045 +/- 663.4	7x
Internode 10	397.8 +/- 123.9	3014 +/- 698.4	8x
Internode 12	441.3 +/- 116.5	3296 +/- 737.6	7x
Internode 14	421.5 +/- 107.8	4047 +/- 1093	10x
Internode 16	414.4 +/- 90.91	3757 +/- 1057	9x
3^rd^ basal Internode	601.5 +/- 155.5	5472 +/- 1287	9x

Except for the tissue cultured plantlets, assays were performed on tissues from maturing plants at the ~20 internode stage of development.

## Discussion

Firefly luciferase is a reporter gene commonly used in promoter functional analysis studies. Firefly luciferase converts the substrate luciferin to oxyluciferin and releases light during the reaction. The light emitted can be detected and quantified using a luminometer or visualised using long exposure cameras. *Renilla* luciferase converts coelenterazine to coelenteramide in a reaction that also emits light. Together, firefly luciferase and *Renilla* luciferase form the basis of a dual luciferase assay that provides researchers with a sensitive and normalised method to quantify promoter activity. However, the application of luciferase genes as reporters of promoter function in sugarcane has been thwarted by aggressive down-regulation and silencing [24].

Synthesising foreign transgene sequences to contain optimized codon usage and G + C contents closer to that of the host species has been an effective way to increase transgene expression in many plant species [1]. Modifying a foreign transgene sequence to mimic coding sequences endogenous to the host plant can be thought of as a ploy to prevent triggering the host plant’s defence mechanisms against foreign gene sequences.

In this study we modified the firefly luciferase and *Renilla* luciferase reporter gene sequences and assessed any enhanced effects on transgene expression in sugarcane. One cautionary note when using firefly luciferase as a reporter in sugarcane comes from the discovery that certain sugarcane tissue extracts inhibit luciferase activity and that some tissue extracts confer stronger inhibition than others [23]. Fortunately, the inhibitory effect can be diluted out and we recommend utilising a 50-100× dilution of tissue extracts (at the concentrations specified in the material and methods) prior to measurement of luciferase activity using a luminometer.

Transient assays in sugarcane suspension cell cultures revealed the synthetic luc* gene produced on average 60-fold higher luciferase activity relative to the native firefly luciferase gene. The synthesized Renluc* gene yielded on average 15-fold higher luciferase activity than the native *Renilla* luciferase gene. Thus, modifying the codon usage of luciferase transgenes to mimic endogenous sugarcane genes and avoiding known destabilizing motifs proved to have a profound positive effect on transient luciferase transgene expression in sugarcane. Such transient assays may provide a fast and efficient preliminary screen for testing the effectiveness of synthetic transgene variants prior to undergoing analysis in stable transgenic lines.

Modifying codon usage has been postulated to increase translational efficiency by avoiding stalling at the ribosome due to uncommon codons encoding rare species of tRNA. However, our design rules also include avoiding polyadenylation signals and known RNA destabilizing motifs. Indeed increasing the G + C content may have also eliminated, by chance, unknown A + T rich RNA destabilizing sequences. Therefore it is of interest to determine if the increase in luciferase activity of our synthesized genes is entirely due to increased translational efficiency or whether our sequence modifications have also increased the stability of the transcript. To compare the steady state transcript levels between the native luciferase genes and our synthesized versions, we developed a novel co-bombardment and qRT-PCR protocol. This transient assay allowed us to detect and quantify ~6.5-fold and ~9-fold increases in the transcript levels derived from both the synthesized Renluc* and luc* transgenes respectively, indicating our sequence modifications have had a positive effect on mRNA steady state levels.

Luciferase assays on populations of stable transgenic sugarcane lines further confirms improved luciferase activity from luc* over the native firefly luciferase gene. luc* activity was significantly higher across each tissue type and stage of development tested. However, it is interesting to note that the fold enhancement of activity by luc* varied considerably from tissue to tissue. An apparent trend that emerged from the data was that fold difference in expression between luc* and luc was progressively stronger in the younger tissues than in the older tissue types tested. It is possible that this sequence-specific tissue-dependent down-regulation of the native firefly luciferase transgene may be due to the action of temporally expressed endogenous regulatory small RNA species such as microRNA’s, for example. Alternatively, silencing mechanisms involving RNA destabilizing motifs, polyadenylation signals or translational inhibition mechanisms may be more pronounced in the younger and more metabolically active tissue types. Other possible explanations include that younger plant tissues have higher metabolic rates which might be reflected in higher transgene expression levels, or that different tissues in different developmental stages may have different populations of tRNA’s or different levels of specific tRNA’s which could potentially alter the codon usage, as has been documented in mammalian systems [38].

The development of synthetic luciferase genes that resist transgene silencing in sugarcane demonstrates the importance of performing careful codon/coding sequence optimization for plant transgene expression, using design rules that have evolved over a number of years from research in various plant species. These optimized luciferase transgenes will likely have great utility across research and applied studies in sugarcane and other monocots. For example, they will benefit future research aimed at characterizing expression patterns conferred by promoters as molecular tools used in basic and applied research. The synthetic luciferase reporter genes could also be used as markers of expression in emerging research fields. For example, we have tagged regulatory small RNA target sequences to the synthetic luc* coding sequence to investigate developmental patterns conferred by small RNAs in sugarcane (Moyle RL, Sternes PR and Birch RG: Incorporating target sequences of developmentally-regulated small RNAs into transgenes to enhance tissue specificity of expression in plants, submitted). These luciferases may also be useful in many dicot plant systems, although this aspect needs to be tested on a case-by-case basis.

The strategy of optimizing transgenes for expression in sugarcane and the molecular techniques developed to quantify enhanced expression characteristics could be applied to many other transgenes of interest in basic and applied sugarcane research. For example, there is much interest in metabolic engineering sugarcane to produce higher sugar yields, healthier sugars and other value-added products [12,28,39].

## Conclusions

In conclusion, we demonstrate that designing transgenes to mimic codon usage in sugarcane and avoid known destabilizing motifs is an effective strategy to optimise transgene expression and prevent transgene silencing in sugarcane. We designed synthetic *Renilla* luciferase and firefly luciferase transgenes that were resistant to silencing in transient assays and in stable transgenic lines. The development of these silencing resistant luciferase reporter genes enables the functional analysis of promoter sequences for use in basic and applied sugarcane research. The application of our strategy to optimise transgene expression and avoid transgene silencing could be applied to other transgenes and advances the prospect of applying genetic engineering strategies to the improvement of sugarcane varieties.

## Methods

### Design and synthesis of luc* and Renluc* coding sequences

Our process for the design of sugarcane optimised synthetic luciferase transgenes used software tools to help eliminate sequence motifs with the potential to cause instability or generate silencing triggers. The design process involved generating sugarcane codon usage tables from the Kazusa database [37]. We also generated a list of specified motifs to be excluded based on potential unintended intron splice signals, known triggers of RNA instability [40] and bioinformatic analysis of rice polyadenylation signals [41] (Additional file 6: Table S1). Candidate sequences were generated using the program Gene Designer [42]. This program excludes codons below our specified threshold value of 20% and then uses a Monte Carlo algorithm based on the probabilities obtained from the codon usage table. Initial candidates were then filtered in an iterative process that attempts to meet additional design criteria including exclusion of the aforementioned motifs and certain DNA repeats, including those generating any mRNA structures with double-stranded RNA stems of 12 bp or more. The program typically was unable to converge on a solution that incorporated all of our specifications as the degeneracy of the excluded motifs in Additional file 6: Table S1 is very demanding. Nonetheless, it provided useful output for further manual refinement to eliminate features of concern such as residual AT-rich strings and repeats. The designed sequences (Additional file 1: Figure S1 and Additional file 2: Figure S2) were synthesised using Genscript Inc (http://www.gencript.com) as the service provider, including two tandem stop codons and flanking *Not*I and *Pac*I restriction sites to facilitate cloning. The synthetic luc* and Renluc* sequences are available in the GenBank database (accession numbers KC147725 and KJ140114, respectively).

### Construct design and preparation

Plasmid was prepared using the plasmid maxiprep kit (QIAGEN) according to the manufacturer’s instructions. The firefly luciferase, *Renilla* luciferase and synthesised luc* and Renluc* coding sequences were cloned into the pUbi T3 background plasmid [28], using unique *Not*I and *Pac*I restriction sites (Figure 1). The pUKR plasmid, containing an nptII kanamycin resistance marker gene under the control of the pUbi promoter [43], was used for selection and for generating negative control lines.

### Dual luciferase assays

Sugarcane suspension cell cultures were prepared for particle bombardment as previously described [44]. Plasmid DNA (5 μg) was coated onto tungsten particles and co-bombarded onto plates of suspension cells as previously described [44]. The bombarded suspension cell plates were incubated in the dark for 24 h. The suspension cells were harvested and ground in the lysis buffer provided in the dual luciferase reporter assay system kit (Promega). Dual luciferase assay reactions were prepared using the dual luciferase reporter assay system (Promega) according to the manufacturer’s instructions. Luciferase activity was quantified using a BMG POLARstar OPTIMA luminometer.

### Quantitative real time PCR

Equimolar quantities of synthesised and native luciferase constructs were co-bombarded onto suspension cell cultures as described above. Co-bombarded suspension cell cultures (100-200 mg) were ground in 1 mL of TRIzol® reagent (Invitrogen) and RNA extracted following the manufacturer’s instructions. Each RNA preparation was treated with 2 units of DNase I (New England Biolabs) at 37°C for 30 min followed by inactivation at 70°C for 10 min. cDNA was synthesized using Oligo(dT)_20_, as previously described [45,46].

qRT-PCR primers were designed to the 3’ region of the target coding sequence (listed in supplementary Figure S3), taking advantage of transgene specific sequence polymorphisms, according to previously described methods [22]. qRT-PCR was performed using FastStart Universal SYBR Green Master (ROX) (Roche Diagnostics) in a Rotorgene 3000 thermocycler (Corbett Research). After denaturation at 95°C for 10 min, the qRT-PCR cycles consisted of denaturation at 95°C for 15 s followed by annealing/extension at 62°C for 60 s for a total of 45 cycles. Differences in expression were calculated using the comparative C_T_ method.

### Generation of stable transgenic lines

Callus initiation from sugarcane cultivar Q117 and callus proliferation was performed as previously described [18]. The aforementioned pUbi-*luc* or pUbi-luc* plasmids were co-bombarded with the selection plasmid pUKR, containing the nptII selection marker under the control of the pUbi promoter, as previously described [18,27]. Lines of transgenic calli were selected, regenerated and grown in the glasshouse as previously described [18]. Negative control plant lines were regenerated from calli bombarded with pUKR only [23].

### Molecular analysis of independent transgenic lines

Multiple plants regenerated from each selected transgenic line of calli were analysed for relative copy number according to the methods described by Basnayake et al. 2011 [18], with the exception of using a different GAPDH reference gene 3’ primer (GAPDHrevb ACGGGATCTCCTCAGGGTTC). In cases where two or more clearly distinct copy numbers were identified among the population of plants regenerated from a single selected transgenic line of calli, those plants were separated and relabelled as independent transgenic lines.

### Luminometer assay of firefly luciferase activity

Transgenic sugarcane tissues were harvested and snap frozen in liquid nitrogen prior to grinding into powder form using a ball mill apparatus (Retsch). Luminometer assays were performed on ground tissue extracts in CCLR* reagent using a BMG POLARstar Optima luminometer [24]. We typically mixed ~15-20 mg of leaf tissue, ~15 mg of meristem-internode 2 tissue, ~50-100 mg of young internode tissue, ~100-200 mg of mature internode tissue and ~100 mg of root tissue with 100 μl of CCLR* to obtain each tissue extract. After thorough mixing, the extracts are centrifuged to pellet insoluble material. As luciferase activity can be inhibited by undiluted sugarcane tissue extracts [23], we typically dilute an aliquot of the soluble fraction of each tissue extract by 100x in CCLR* reagent prior to performing luciferase assay measurements in a luminometer. The output measurement readings from the luminometer are referred to as the relative light units (RLU). Bradford assays using protein assay dye reagent (Bio-Rad) were utilised to quantify the soluble protein concentration within the soluble fraction of each undiluted tissue extract [47].

### *In vivo* firefly luciferase activity camera assay

Luciferase activity in plated and bombarded suspension cells or transverse internode sections was imaged by first immersing samples in 0.4 mM luciferin as previously described [44,48]. The light emission was measured using a PIXIS 102A camera over a 500 second exposure time. WinView32 software (Princeton Instruments) was used to measure the average pixel intensities (background subtracted) across each internode section.

### Statistical analysis

Luciferase activity data were log transformed to more closely approximate the mathematical assumption of normality and equality variance in the experimental data, and analysed using GraphPad Prism 5 software.

## Competing interests

The authors declare they have no competing interests.

## Authors’ contributions

TCC carried out transgenic and molecular analyses. RLM designed the study, contributed to the molecular and computational analyses, and drafted the manuscript. Each author read and approved the final manuscript.

## Supplementary Material

Additional file 1: Figure S1 Alignment between the native firefly *luc* coding and the synthetic luc* coding sequences. Base changes between the native firefly luciferase and the synthesized version are shaded. The synthesized luc* coding sequence shares 75% identity with the native *luc* coding sequence. Click here for file

Additional file 2: Figure S2 Alignment between the Ren*luc* coding sequence and the silencing resistant synthetic Renluc* sequence. Base changes between the native *Renilla* luciferase and the synthesized **version** are shaded. The synthesized Renluc* coding sequence shares 75% identity with the native Ren*luc* sequence. Click here for file

Additional file 3: Figure S3 Camera assay on sugarcane suspension cell cultures bombarded with either pUbi-*luc* or pUbi-luc*. Equi-molar amounts of pUbi-*luc* or pUbi-luc* were bombarded at replicate plates of sugarcane suspension cells. The plates were incubated for 24 hrs, saturated in luciferin and the resulting light emission visualised under long exposure using a PIXIS 102A camera. Click here for file

Additional file 4: Figure S4 Primer pairs used for qRT-PCR and the amplicons generated from each luciferase template sequence. The underlined bases in the primer sequences are transgene specific base pairs. **A**. Primer sequences designed to *luc* and the resulting amplicon. **B**. Primer sequences designed to luc* and the resulting amplicon. **C**. Primer sequences designed to Ren*luc* gene and the resulting amplicon. **D**. Primer sequences designed to the Renluc* gene and the resulting amplicon. Click here for file

Additional file 5: Figure S5 Transgene copy numbers of pUbi-*luc* and p-Ubi-luc* populations of lines. The error bars represent the standard error of the mean across 15 transgenic lines per construct. Click here for file

Additional file 6: Table S1 Excluded motifs for computer-aided optimization of transgenes. Click here for file
