# Expression of concern: Synthetic versions of firefly luciferase and Renilla luciferase reporter genes that resist transgene silencing in sugarcane

**DOI:** 10.1186/s12870-015-0671-6

**Published:** 2015-12-30

**Authors:** Jigisha Patel, Maria Kowalczuk

**Affiliations:** The authors of this Expression of Concern are members of The Research Integrity Group at BioMed Central. BioMed Central, 236 Gray’s Inn Road, London, WC1X 8HB UK

## Expression of concern

The publishers of this journal are publishing this Expression of Concern because attribution for the synthesis and design of the luc* and Renluc* coding sequences in this article [1] is under dispute.

The correct table and description of the method set out in Table S1 can be found in this article [2].

The University of Queensland advises that there has been no academic misconduct but the wording of any erratum remains under dispute.
