# Supplementary figures and images for: Synthetic versions of firefly luciferase and Renilla luciferase reporter genes that resist transgene silencing in sugarcane
Source: BMC Plant Biol. 2014 Apr 8;14:92. doi: 10.1186/1471-2229-14-92 (PMC4021088; doi:10.1186/1471-2229-14-92)

## Slide 1
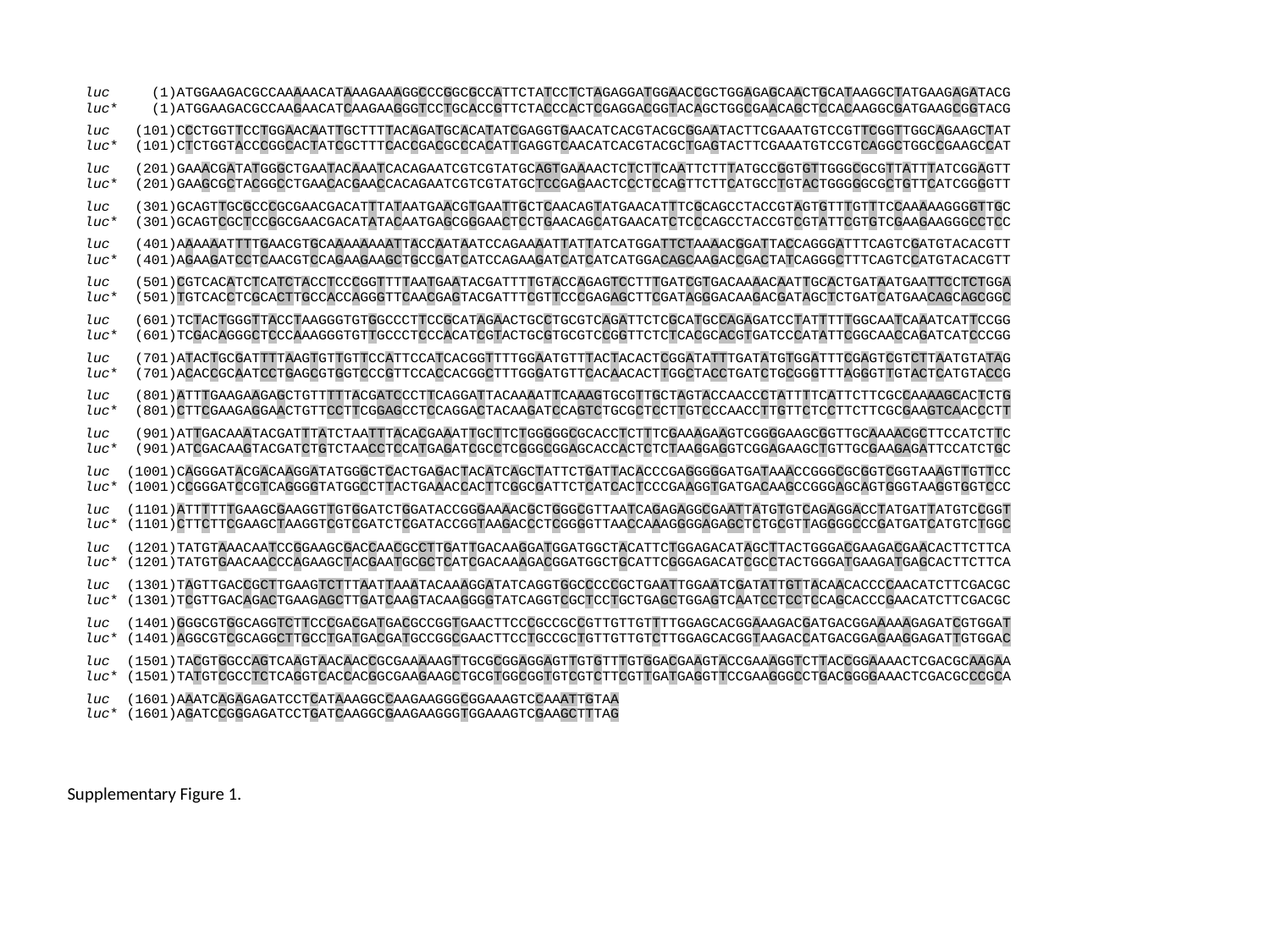

Supplementary Figure 1.

Supplement: Additional file 1: Figure S1 — Alignment between the native firefly luc coding and the synthetic luc* coding sequences. Base changes between the native firefly luciferase and the synthesized version are shaded. The synthesized luc* coding sequence shares 75% identity with the native luc coding sequence. [file 1471-2229-14-92-S1.pptx]

## Slide 1
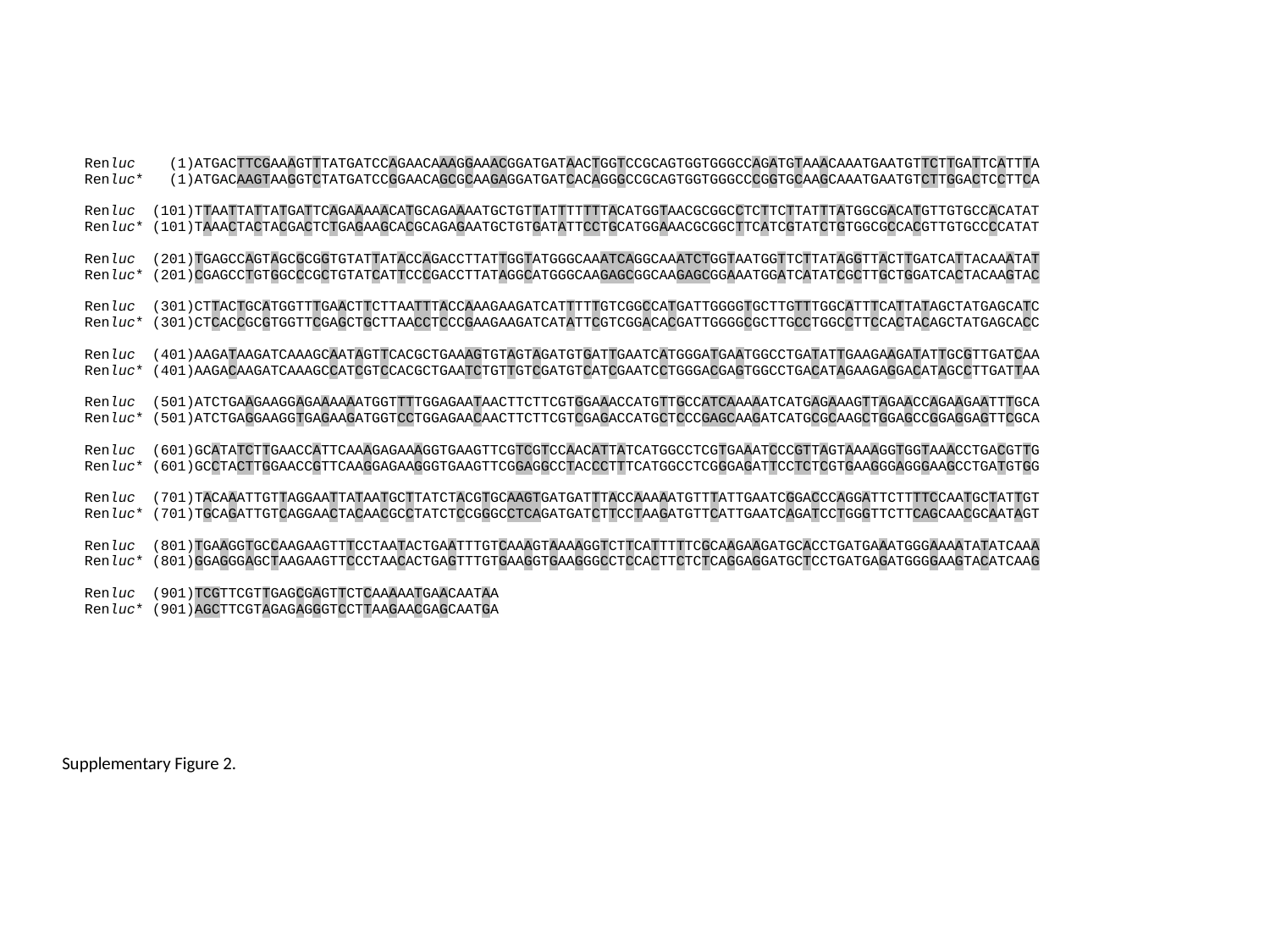

Supplementary Figure 2.

Supplement: Additional file 2: Figure S2 — Alignment between the Renluc coding sequence and the silencing resistant synthetic Renluc* sequence. Base changes between the native Renilla luciferase and the synthesized version are shaded. The synthesized Renluc* coding sequence shares 75% identity with the native Renluc sequence. [file 1471-2229-14-92-S2.pptx]

## Slide 1
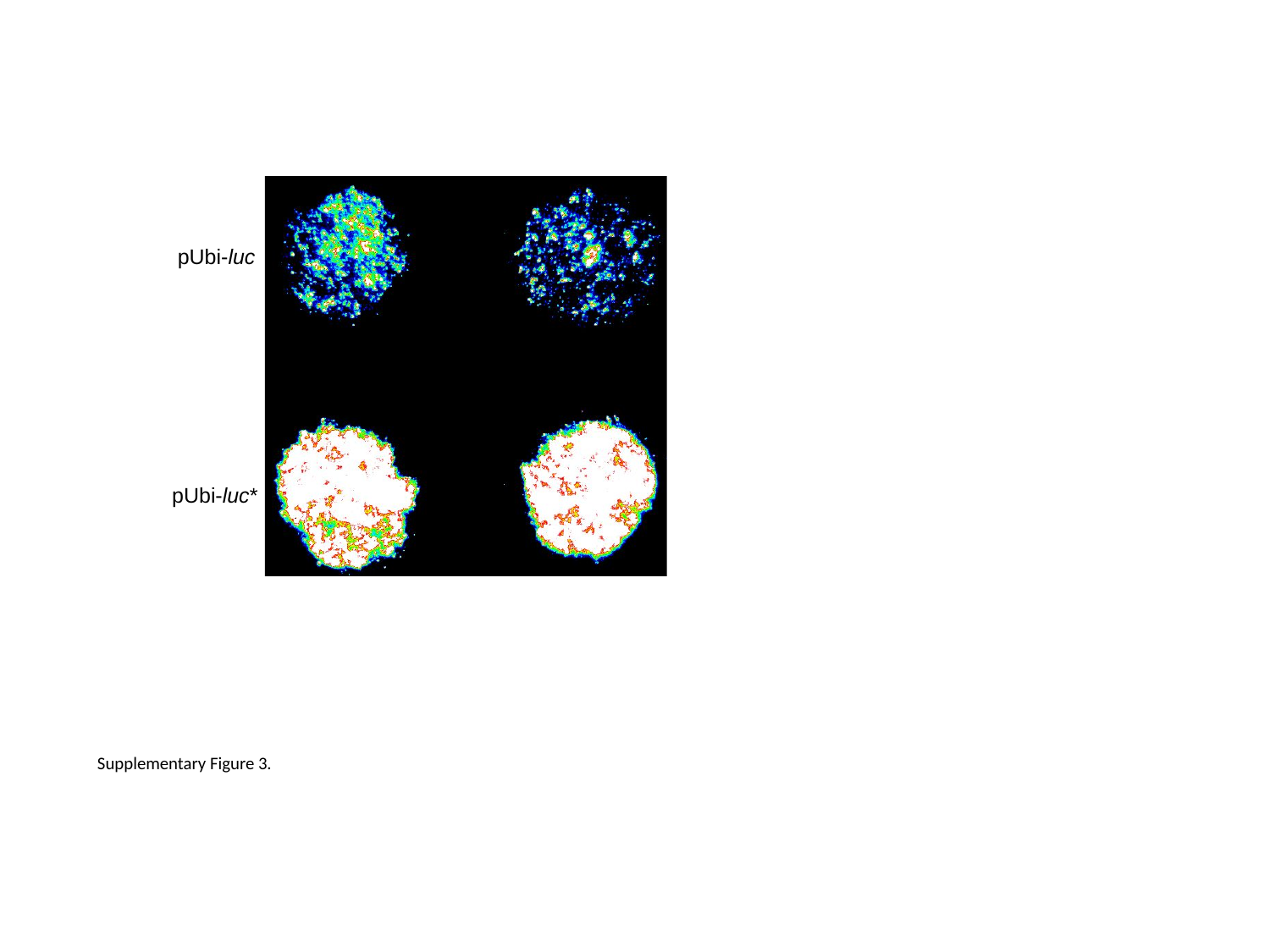

pUbi-luc
pUbi-luc*
Supplementary Figure 3.

Supplement: Additional file 3: Figure S3 — Camera assay on sugarcane suspension cell cultures bombarded with either pUbi-luc or pUbi-luc*. Equi-molar amounts of pUbi-luc or pUbi-luc* were bombarded at replicate plates of sugarcane suspension cells. The plates were incubated for 24 hrs, saturated in luciferin and the resulting light emission visualised under long exposure using a PIXIS 102A camera. [file 1471-2229-14-92-S3.pptx]

## Slide 1
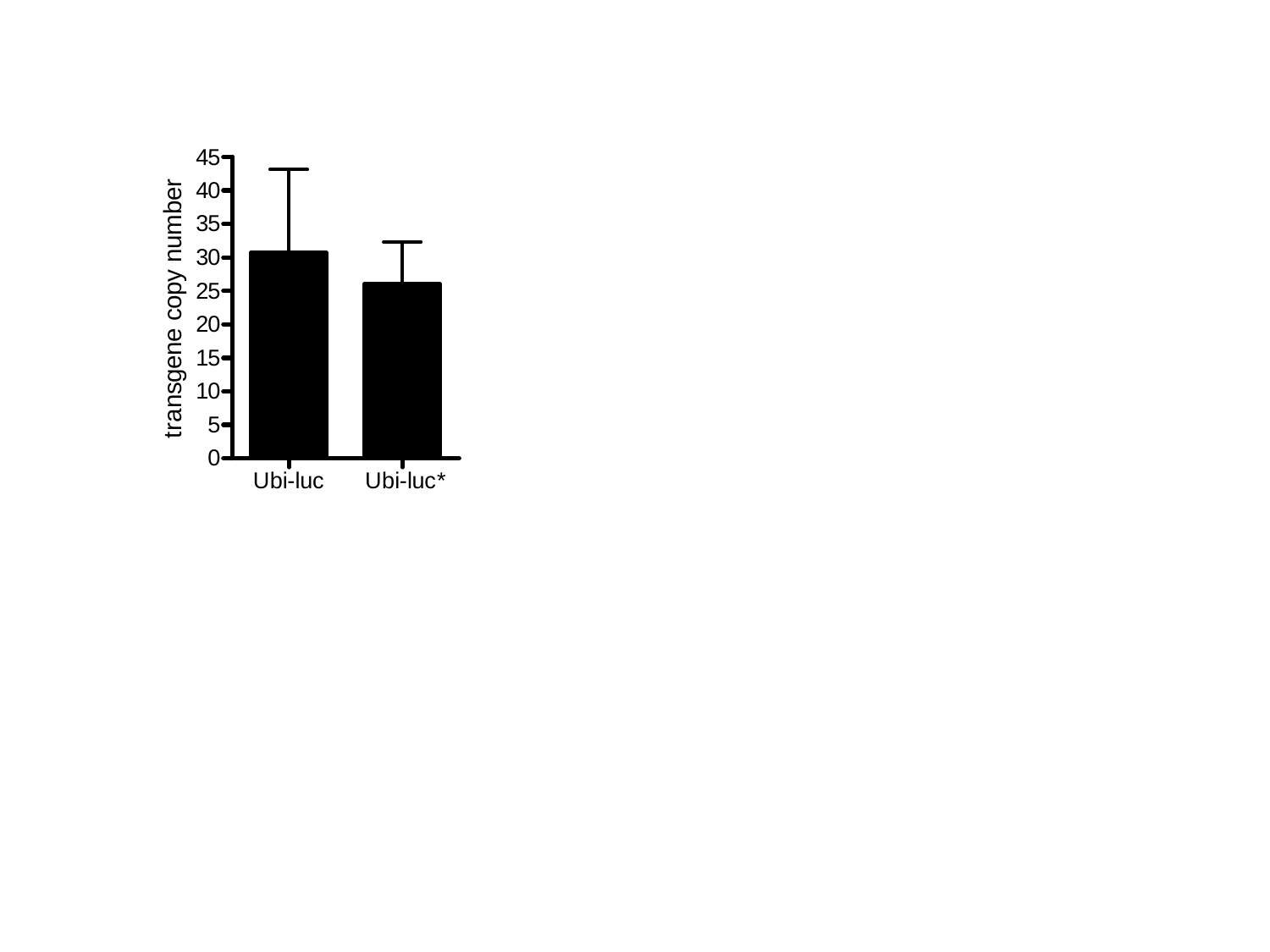

Supplement: Additional file 5: Figure S5 — Transgene copy numbers of pUbi-luc and p-Ubi-luc* populations of lines. The error bars represent the standard error of the mean across 15 transgenic lines per construct. [file 1471-2229-14-92-S5.pptx]
