# Supplementary material for: Synthetic versions of firefly luciferase and Renilla luciferase reporter genes that resist transgene silencing in sugarcane
Source: BMC Plant Biol. 2014 Apr 8;14:92. doi: 10.1186/1471-2229-14-92 (PMC4021088; doi:10.1186/1471-2229-14-92)
Supplement: Additional file 4: Figure S4 — Primer pairs used for qRT-PCR and the amplicons generated from each luciferase template sequence. The underlined bases in the primer sequences are transgene specific base pairs. A. Primer sequences designed to luc and the resulting amplicon. B. Primer sequences designed to luc* and the resulting amplicon. C. Primer sequences designed to Renluc gene and the resulting amplicon. D. Primer sequences designed to the Renluc* gene and the resulting amplicon. [file 1471-2229-14-92-S4.pptx]

## Slide 1
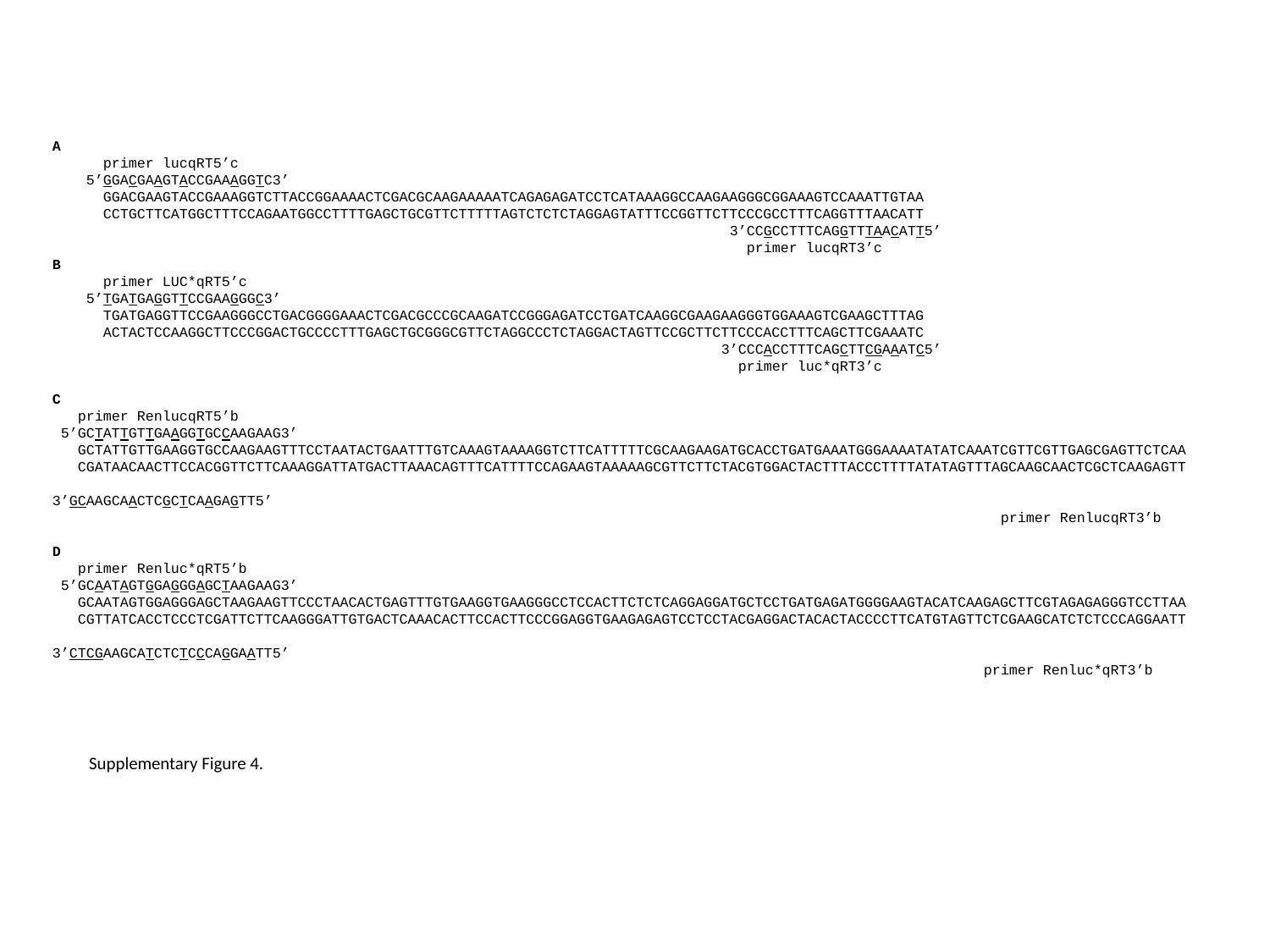

A
 primer lucqRT5’c
 5’GGACGAAGTACCGAAAGGTC3’
 GGACGAAGTACCGAAAGGTCTTACCGGAAAACTCGACGCAAGAAAAATCAGAGAGATCCTCATAAAGGCCAAGAAGGGCGGAAAGTCCAAATTGTAA
 CCTGCTTCATGGCTTTCCAGAATGGCCTTTTGAGCTGCGTTCTTTTTAGTCTCTCTAGGAGTATTTCCGGTTCTTCCCGCCTTTCAGGTTTAACATT
 3’CCGCCTTTCAGGTTTAACATT5’
 primer lucqRT3’c
B
 primer LUC*qRT5’c
 5’TGATGAGGTTCCGAAGGGC3’
 TGATGAGGTTCCGAAGGGCCTGACGGGGAAACTCGACGCCCGCAAGATCCGGGAGATCCTGATCAAGGCGAAGAAGGGTGGAAAGTCGAAGCTTTAG
 ACTACTCCAAGGCTTCCCGGACTGCCCCTTTGAGCTGCGGGCGTTCTAGGCCCTCTAGGACTAGTTCCGCTTCTTCCCACCTTTCAGCTTCGAAATC
 3’CCCACCTTTCAGCTTCGAAATC5’
 primer luc*qRT3’c
C
 primer RenlucqRT5’b
 5’GCTATTGTTGAAGGTGCCAAGAAG3’
 GCTATTGTTGAAGGTGCCAAGAAGTTTCCTAATACTGAATTTGTCAAAGTAAAAGGTCTTCATTTTTCGCAAGAAGATGCACCTGATGAAATGGGAAAATATATCAAATCGTTCGTTGAGCGAGTTCTCAA
 CGATAACAACTTCCACGGTTCTTCAAAGGATTATGACTTAAACAGTTTCATTTTCCAGAAGTAAAAAGCGTTCTTCTACGTGGACTACTTTACCCTTTTATATAGTTTAGCAAGCAACTCGCTCAAGAGTT
 3’GCAAGCAACTCGCTCAAGAGTT5’
 primer RenlucqRT3’b
D
 primer Renluc*qRT5’b
 5’GCAATAGTGGAGGGAGCTAAGAAG3’
 GCAATAGTGGAGGGAGCTAAGAAGTTCCCTAACACTGAGTTTGTGAAGGTGAAGGGCCTCCACTTCTCTCAGGAGGATGCTCCTGATGAGATGGGGAAGTACATCAAGAGCTTCGTAGAGAGGGTCCTTAA
 CGTTATCACCTCCCTCGATTCTTCAAGGGATTGTGACTCAAACACTTCCACTTCCCGGAGGTGAAGAGAGTCCTCCTACGAGGACTACACTACCCCTTCATGTAGTTCTCGAAGCATCTCTCCCAGGAATT
 3’CTCGAAGCATCTCTCCCAGGAATT5’
 primer Renluc*qRT3’b
Supplementary Figure 4.
