# Supplementary material for: Synthetic versions of firefly luciferase and Renilla luciferase reporter genes that resist transgene silencing in sugarcane
Source: BMC Plant Biol. 2014 Apr 8;14:92. doi: 10.1186/1471-2229-14-92 (PMC4021088; doi:10.1186/1471-2229-14-92)
Supplement: Additional file 6: Table S1 — Excluded motifs for computer-aided optimization of transgenes. [file 1471-2229-14-92-S6.docx]

**Supplementary Table 1. Excluded motifs for computer-aided optimization of transgenes.**

| **Description** | **Sequence** | **Comments** |
| --- | --- | --- |
| Cryptic splice donor | MAGGTRAGT | M = A or C; R = A or G |
| Cryptic splice acceptor | YYYYNTAGG | Y = C or T; N = A,T,C or G |
| RNA destabilizing sequence | ATTTA |  |
| NUE1 | AATAAA |  |
| NUE2 | WWWWWW | W = A or T |
| FUE-TRE-CE | TTTT |  |
| FUE-ORY1 | WWWWGT | W = A or T |
| FUE-ORY2 | GTGTG |  |
| FUE-ORY3 | TGTAW | W = A or T |
| FUE-ORY4 | WTGTA | W = A or T |
| ATRICH1 | WSWWWW | W = A or T; S = C or G |
| ATRICH2 | WWSWWW | W = A or T; S = C or G |
| ATRICH3 | WWWSWW | W = A or T; S = C or G |
